# Iberverin exhibits antineoplastic activities against human hepatocellular carcinoma via DNA damage-mediated cell cycle arrest and mitochondrial-related apoptosis

**DOI:** 10.3389/fphar.2023.1326346

**Published:** 2023-12-13

**Authors:** Yuting Zhang, Jiao Du, Libo Jin, Liying Pan, Xiufeng Yan, Sue Lin

**Affiliations:** ^1^ College of Life and Environmental Science, Wenzhou University, Wenzhou, Zhejiang, China; ^2^ Institute of Life Sciences, Wenzhou University, Wenzhou, Zhejiang, China; ^3^ Zhejiang Provincial Key Laboratory for Water Environment and Marine Biological Resources Protection, Wenzhou University, Wenzhou, Zhejiang, China

**Keywords:** hepatocellular carcinoma, iberverin, natural compound, antineoplastic activity, apoptosis, G2/M arrest

## Abstract

Hepatocellular carcinoma (HCC) is one of the malignant tumors with high incidence and mortality rates in the world. Isothiocyanates (ITCs), bioactive substances present primarily in the plant order Brassicales, have been proved to be promising candidates for novel anti-HCC drugs with chemopreventive and anticancer activities. Iberverin, a predominant ITC isolated from the seeds of oxheart cabbage, has been discovered with anticancer property in lung cancer cells. However, the roles of iberverin in HCC remain elusive. In the present study, the effect and potential mechanisms of iberverin against human HCC were dissected. We demonstrated that low concentrations of iberverin inhibited cell proliferation, suppressed migration and induced mitochondrial-related apoptosis *in vitro*, and hampered tumorigenicity *in vivo*, with no obvious toxicity. Furthermore, we found that iberverin treatment induced DNA damage and G2/M phase arrest. Iberverin treatment also caused increased intracellular reactive oxygen species formation and glutathione depletion. Taken together, these results suggest that iberverin promotes mitochondrial-mediated apoptosis and induces DNA damage and G2/M cell cycle arrest in HCC by enhancing oxidative stress. Our findings provide better understanding of the anti-HCC mechanisms of ITCs and the potential for the natural product iberverin as a promising new anti-HCC biotherapeutic.

## 1 Introduction

Liver cancer is the sixth most common cancer and also the third leading cause of cancer-related deaths in the world, among which hepatocellular carcinoma (HCC) is the most common pathological type accounting for −90% of cases (Llovet et al., 2016; Siegel et al., 2021; Sung et al., 2021). Considerable evidence demonstrates that the incidence of HCC is mostly associated with chronic liver disease, hepatitis virus B (HBV) and C (HCV) infection, and unhealthy drinking (Lin et al., 2013; Trinchet et al., 2015; Akinyemiju et al., 2017; Estes et al., 2018). Due to the relatively insidious onset and the lack of early screening, most HCC patients are frequently at advanced stages at the time of diagnosis and not eligible for curative surgery (Singh et al., 2014). For these patients, promising therapeutic strategies such as targeted drug therapy and systemic chemotherapy are the pivotal options. However, clinical application of these strategies, even treatments with sorafenib, lenvatinib or the combination of atezolizumab and bevacizumab, also cause some adverse reactions, such as elevated aspartate aminotransferase, hypertension and proteinuria (Llovet et al., 2021).

Biologically active natural compounds and their derivatives have drawn growing attention and have been confirmed as one of the most feasible solutions for hard-to-treat cancers (Dong et al., 2020; Fontana et al., 2020; Kubczak et al., 2021; Tewari et al., 2022). Isothiocyanates (ITCs) are hydrolysis products derived from glucosinolates, one of the plant secondary metabolites in the order Brassicales (Nambiar et al., 2020). Accumulating evidence from encouraging *in vitro* and *in vivo* animal models indicates that several ITCs exert chemopreventive activity and inhibitory effects on multiple cancers, such as lung cancer, oral cancer and liver cancer (Rakariyatham et al., 2019; Boldry et al., 2020; Rekha et al., 2022; Zhang et al., 2022). Iberverin, namely, 3-methylthiopropyl ITC, is a predominant ITC in the seeds of oxheart cabbage (*Brassica oleracea* var. *capitata*) (Wang et al., 2010). It has been previously proved to possess anticancer properties in A549 lung carcinoma cells by promoting apoptosis through activation of Caspase-3,-8 and -9 (Wang et al., 2016). However, the potential roles of iberverin in HCC and its molecular mechanism remain elusive.

In this study, *in vitro* and *in vivo* assays were performed to investigate the effect and potential mechanisms of iberverin on HCC. It has been found that iberverin could inhibit cell proliferation, suppress invasion and migration abilities, promote mitochondrial-related apoptosis, and arrest cell cycle in G2/M-phase arrest in HCC in a dose-dependent manner. We demonstrated that iberverin-induced cell cycle arrest could be associated with DNA damage mediated by the induction of oxidative stress via accumulating reactive oxygen species (ROS) and consuming reduced glutathione (GSH). Meanwhile, iberverin could effectively inhibit the tumorigenic ability of HCC cells in mice with no obvious toxic effect on the body. These results suggest that the natural product iberverin has become a potentially promising new anti-HCC biotherapeutic.

## 2 Materials and methods

### 2.1 Cell lines and reagents

The human HCC cell lines HCCLM3, HepG2, Huh7, Huh7.5.1 and SMMC7721 were purchased from the China Typical Culture Collection Center (CCTCC), SNU739 from the Korean Cell Line Bank (KCLB), and Huh1 from the Japanese Collection of Research Bioresources Cell Bank (JCRB). Cell lines HCCLM3, Huh1, Huh7, Huh7.5.1 and SMMC7721 were cultured in dulbecco’s modified eagle medium (DMEM) (Gibco, the United States, catalog no. C11995500BT) supplemented with 10% fetal bovine serum (FBS) (Biological Industries, Israel, catalog no. 04-001-1ACS) and 1% v/v penicillin-streptomycin (Solarbio, P. R. China, catalog no. P1400). Cell lines SNU739 and HepG2 were cultured in RPMI 1640 medium (Gibco, the United States, catalog no. C11875500BT) and MEM (Procell, P. R. China, catalog no. PM150410), respectively, supplemented with 10% FBS and 1% v/v penicillin-streptomycin. Cells were incubated at 37°C with 5% CO_2_. All cell lines were negative for *mycoplasma* contamination.

Purified iberverin (>98%) was purchased from aladdin (Aladdin, P. R. China, catalog no. 505-79-3), dissolved in dimethyl sulfoxide (DMSO) (Solarbio, P. R. China, catalog no. D8371) to prepare a 500 mmol/L stock solution, and then stored in dark at −80°C.

### 2.2 MTT assay and colony formation assay

The antiproliferative effect of iberverin on human HCC cells was evaluated by MTT assay. HCCLM3, HepG2, Huh1, Huh7, Huh7.5.1, SMMC7721 and SNU739 cells were harvested and seeded in triplicate in a 96-well plate (5 × 10^3^ cells per well) and incubated overnight. After 48 h of drug regimen treatments, 30 µL of MTT solution (Solarbio, P. R. China, catalog no. 298-93-1) was added into each well. After 4 h of incubation at 37°C, the formazan crystals were dissolved in 150 µL of DMSO, and the absorbance was measured at 490 nm with a microplate spectrometer (SPARK, Tecan, Swiss). The half maximal inhibitory concentration (IC_50_) values were calculated using GraphPad Prism version 9.0.

For the colony formation assay, Huh7, Huh7.5.1 and SNU739 cells were seeded into a 6-well plate at a density of 5000 cells per well and incubated with 10, 20, and 40 μmol/L iberverin or 0.1% DMSO control for 12 h. After incubation in fresh medium for 2 weeks, the cell colonies were washed twice with phosphate buffered solution (PBS) (Gibco, the United States, catalog no. C20012500BT), fixed with 4% paraformaldehyde (Biosharp, P. R. China, catalog no. BL539A) at room temperature for 15 min, washed three times with PBS, and stained with crystal violet (Beyotime, P. R. China, catalog no. C0121) for 15 min. The stained colonies were counted using ImageJ software.

### 2.3 Transwell invasion assay and wound healing migration assay

For wound healing migration assay, Huh7, Huh7.5.1 and SNU739 cells were seeded into 6-well plates (10^5^ cells per well). When cells reached 95%–100% confluence, a cell-free gap was created after scratching with a 10 μL sterile pipette tip. After washing with PBS to remove detached cells, cells were treated with 10 μmol/L iberverin or DMSO control for 12 h. At 0, 24, 48, and 72 h, the images were captured with a bright field microscope (Nikon, TS2-S-SM, Japan), and the migration distance was analyzed by ImageJ software.

For transwell invasion assay, Huh7, Huh7.5.1 and SNU739 cells were seeded into the upper chamber of 24-well transwell plates at a density of 2 × 10^4^ cells per well, and 600 μL of medium with 10% FBS was added to the lower chamber. After coculture with 10, 20 and 40 μmol/L iberverin or DMSO control for 12 h, cells that adhered to the lower surface were washed with PBS, fixed with 4% paraformaldehyde at room temperature for 15 min, washed three times with PBS, and stained with crystal violet for 15 min. The migrated cells on the lower surface were photographed with a bright field microscope (Nikon, TS2-S-SM, Japan).

### 2.4 Apoptosis assay by flow cytometry and TUNEL assay

Apoptosis assay was performed with a Annexin V-FITC/PI apoptosis detection kit (BD, the United States, catalog no. 556547) to measure iberverin-induced apoptosis in HCC cells. Briefly, after incubation with 20 and 40 μmol/L iberverin or DMSO control for 12 h, Huh7, Huh7.5.1, and SNU739 cells were collected and washed twice with PBS. Then, cells were resuspended with 400 μL of binding buffer, and stained with 5 μL of Annexin V conjugated with fluorescein isothiocyanate (FITC) and 5 μL of propidium iodide (PI) solution to each sample. After incubation in dark for 15 min at room temperature, samples were analyzed by flow cytometry (FCM) (NovoCyte, Agilent, the United States).

A terminal deoxynucleotidyl transferase-mediated dUTP nick-end labeling (TUNEL) assay was conducted with a One Step TUNEL Apoptosis Assay Kit (Beyotime, P. R. China, catalog no. C1088) according to the manufacturer’s instructions in conjunction with the DAPI staining to detect DNA fragmentation induced by iberverin in HCC cells. In brief, Huh7, Huh7.5.1 and SNU739 cells were seeded in 6-well plates (10^4^ cells per well), cultured overnight, and then incubated with 20 and 40 μmol/L iberverin or DMSO control for 12 h. Subsequently, cells were washed with PBS and then fixed with 4% paraformaldehyde for 30 min. After infiltration with Triton X-100 (Beyotime, P. R. China, catalog no. P0096) for 5 min, cells were incubated with TUNEL reaction buffer for 30 min at 37°C in dark. The nuclei of cells were further stained with 10 μL of 5 mg/L DAPI solution (BBI, P. R. China, catalog no. E607303) for 20 min in dark. Samples were observed under a fluorescence microscopy (Nikon, TS2-S-SM, Japan) to view the green fluorescence of apoptotic cells at 520 nm and blue DAPI-stained nuclei at 460 nm.

### 2.5 Detection of Caspase-3 activity

Huh7, Huh7.5.1 and SNU739 cells were seeded into 6-well plates at a density of 10^5^ cells per well and cultured overnight. After incubation with 20 and 40 μmol/L iberverin or DMSO control for 12 h, Caspase-3 enzymatic activity level was measured via the detection of *p*-nitroaniline (*p*NA) cleavage by Caspase-3-specific substrates using a Caspase-3 assay kit (Beyotime, P. R. China, catalog no. C1116). According to the manufacturer’s instructions, cells were collected, lysed and then centrifuged at 20,000 × g for 15 min at 4°C in a precooled centrifuge. The enzymatic reaction for Caspase-3 activity was performed at 37°C for 2 h with 50 μL of cell lysate, 40 μL of reaction buffer and 10 μL of Caspase-3 colorimetric substrate (acetyl-Asp-Glu-Val-Asp *p*-nitroanilide, Ac-DEVD-*p*NA) in each reaction. The color intensity of *p*NA was measured at 405 nm with a microplate spectrometer (SPARK, Tecan, Swiss).

### 2.6 Determination of mitochondrial membrane potential

Huh7, Huh7.5.1 and SNU739 cells were seeded into 6-well plates (10^5^ cells per well) and cultured overnight. After incubation with 20 and 40 μmol/L iberverin or DMSO control for 12 h, cells were collected and washed with PBS. The changes in mitochondrial membrane potential (MMP) were detected using the JC-1 Assay Kit (Beyotime, P. R. China, catalog no. C2006) under a fluorescence microscopy (Nikon, TS2-S-SM, Japan) as described in the product manual.

### 2.7 Cell cycle analysis

Huh7, Huh7.5.1 and SNU739 cells were seeded into 6-well plates (10^5^ cells per well) and cultured overnight. After incubation with 20 and 40 μmol/L iberverin or DMSO control for 12 h, cells were harvested, washed twice with precooled PBS and stored in 75% ethanol at −20°C overnight. For PI/RNase staining, overnight-fixed cells were resuspended with 500 μL of PI/RNase Staining Buffer (BD, the United States, catalog no. 550825). After incubation away from light for 15 min at room temperature, samples were analyzed by FCM.

### 2.8 Western blotting and antibodies

After treatment with 20 μmol/L iberverin, the total cellular protein was extracted using the RIPA lysis buffer (Boster, P. R. China, catalog no. AR0103) and separated by 12.5% SDS-PAGE gel (YEASEN, P.R. China, catalog no. 20326ES62). Target protein was then transferred from the SDS-PAGE gel to a PVDF membrane (Merck Millipore, the United States, catalog no. IPVH00010) followed by blocking with 5% skim milk (Solarbio, P. R. China, catalog no. LP0033B). The membranes were incubated overnight at 4°C with the corresponding primary antibodies, washed, and then incubated with the proper secondary antibodies. GAPDH was used for normalization. Anti-Bax (CST, the United States, catalog no. 2772S) and anti-GAPDH (CST, the United States, catalog no. 2118S) were purchased from Cell Signaling Technology (Beverly, MA, the United States). Anti-Bcl-2 (BBI, P. R. China, catalog no. D151534) was purchased from BBI (China).

### 2.9 Determination of oxidative stress and antioxidant defenses

Huh7, Huh7.5.1, and SNU739 cells were seeded into 6-well plates and cultured overnight. After incubation with 20 and 40 μmol/L iberverin or DMSO control for 12 h, cells were harvested. For ROS assay, the oxidation-sensitive fluorescent probe DCFH–DA (Beyotime, P. R. China, catalog no. S0033S) was used to detect intracellular ROS levels according to the instructions by FCM. For quantification of GSH, a GSH assay kit (Solarbio, P. R. China, catalog no. BC1175) was used according to the manufacturer’s instructions. The GSH concentration was normalized to the cell number.

### 2.10 Nude mice xenograft assay

A total of ten immunodeficient BALB/c nude female mice (4–6 weeks-old) purchased from Hangzhou Medical College were randomly divided into two groups with five mice in each group and allowed to adapt for 1 week prior to the experiment. Huh7.5.1 cells (1 × 10^7^) were implanted into the back of mice by subcutaneous injection. At 7 days after implantation, mice were treated as follows: 1) control group, 50 μL of DMSO, intraperitoneal injection; 2) 20 mg/kg iberverin dissolved in DMSO, intraperitoneal injection. The treatment regimens were administered every 3 days for five cycles, and the tumor size and the body weight of mice were measured every 3 days. After the animals were sacrificed under anesthesia, the tumor tissues were removed and weighted. The tumor volume calculation formula was as follows: V = 0.5 × length × width^2^. All animal procedures were in accordance with the guidelines of the Animal Policy and Welfare Committee of Wenzhou University.

### 2.11 Histopathology and immunohistochemistry staining

Tissue samples were fixed with 4% paraformaldehyde, embedded in paraffin, and cut into 4 μm sections. Paraffin sections of organs were stained with hematoxylin-eosin (H&E) staining for routine histological examination and morphometric analysis. For immunohistochemistry (IHC) staining, tumor sections were deparaffinized with xylene and gradually rehydrated with ethanol, followed by incubation in 3% hydrogen peroxide to block the endogenous peroxidase activity. After heat-mediated antigen retrieval, the slides were blocked with 10% normal goat serum for 15 min, and then incubated with anti-Ki-67 (Abcam, the United Kingdom, catalog no. ab16667), anti-PCNA (Proteintech, the United States, catalog no. 10205-2-AP) and anti-Cleaved Caspase-3 (Cell Signaling, the United States, catalog no. 9661s) antibodies, respectively, at 4°C overnight. The sections were washed with PBS and incubated with the secondary antibodies at room temperature for 1 h, followed by detection using a DAB kit (DAKO, Denmark, catalog no. K5007) and examination by light microscopy.

### 2.12 RNA-seq and data analysis

A total of 1 μg RNA was isolated from Huh7, Huh7.5.1 and SNU739 cells treated with 20 μmol/L iberverin or DMSO control for 12 h, respectively, and then enriched with polyA^+^ using Dynabeads Oligo (dT) magnetic beads (Thermo Fisher, the United States, catalog no. 25-61005). The polyA^+^ RNA was used as input material for library preparations using a NEBNext^®^ Magnesium RNA Fragmentation Module (NEB, the United States, catalog no. E6150S). Subsequently, paired-end sequencing was performed on Illumina NovaseqTM 6000 by LC-Bio Technology Co., Ltd. (Hangzhou, China). Gene expression abundance was showed by Fragments per Kilobase of Transcript per Million mapped reads (FPKM). Gene Set Enrichment Analysis (GSEA) was performed for gene functional annotation.

### 2.13 Real-time RT-PCR analysis

Residual RNA samples for RNA-seq were transcribed into cDNA using a PrimeScript™ RT reagent kit with gDNA Eraser (TAKARA, Japan, catalog no. RR047A). *GAPDH* was used as the normalization control. Real-time RT-PCR was performed on a BioRad CFX Connet Real-time RT-PCR Dectction system (Bio-Rad, the United States) and the relative expression levels were analyzed. All primers used were listed in Supplementary Table S1.

### 2.14 Statistical analysis

Statistical differences between groups were determined using *t*-test or one-way ANOVA using GraphPad Prism version 9.0. *p*-values <0.05 were considered statistically significant.

## 3 Results

### 3.1 Iberverin inhibits the viability and proliferation of HCC cells *in vitro*

**FIGURE 1:**
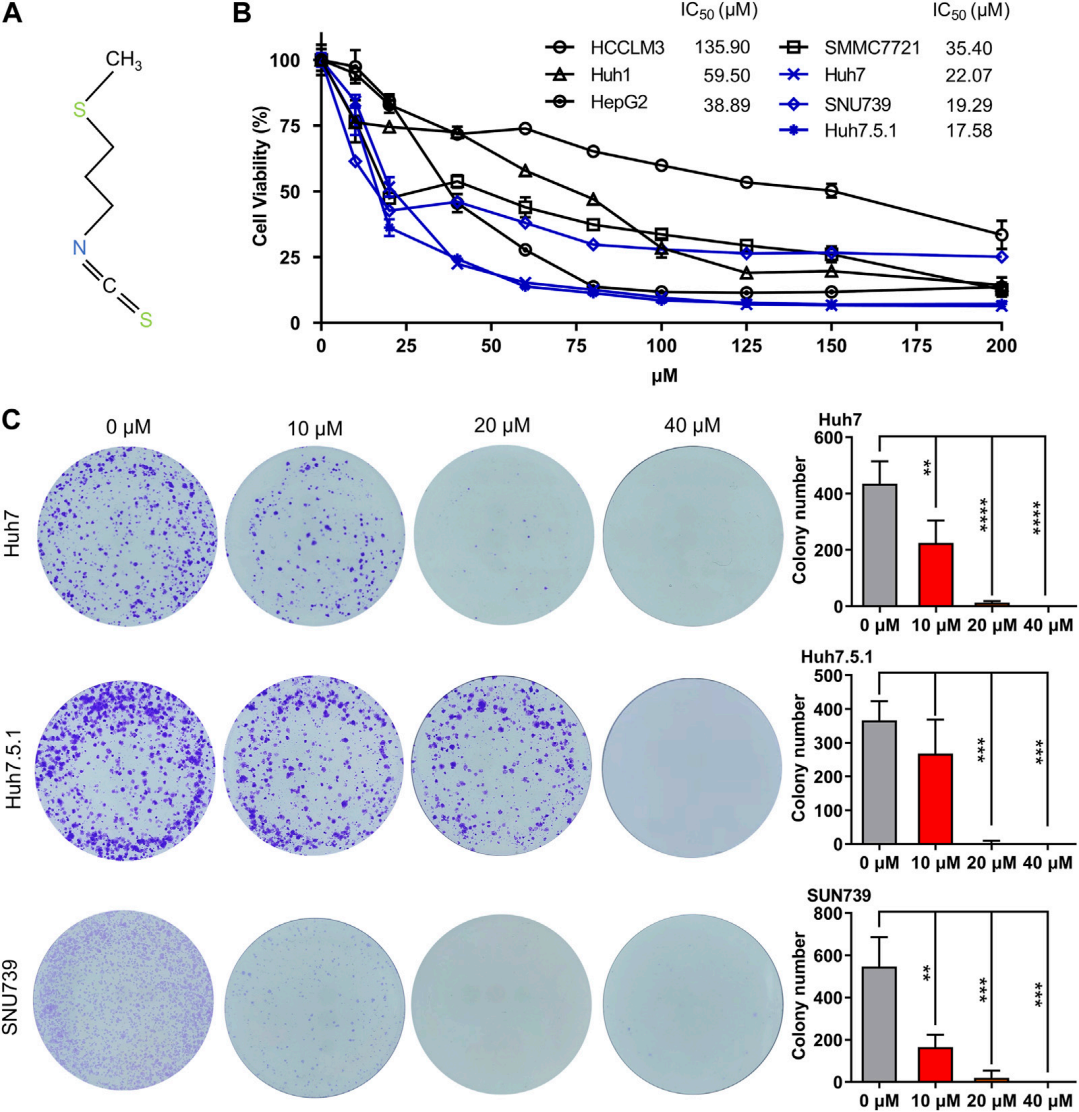
Iberverin inhibits the viability and proliferation of HCC cells. **(A)** The chemical structure formula of iberverin. **(B)** HCCLM3, HepG2, Huh1, Huh7, Huh7.5.1, SMMC7721 and SNU739 cells were incubated with iberverin for 48 h. The 50% inhibiting concentrations (IC_50_) were calculated based on the MTT assay (*n* = 3). **(C)** Colony formation assay of Huh7, Huh7.5.1 and SNU739 cells incubated with 10, 20, and 40 μmol/L iberverin or DMSO control for 2 weeks (*n* = 3). Data were shown as mean ± SD (standard deviation). **p < 0.01, ***p < 0.001 and ****p < 0.0001.

In order to demonstrate the broad inhibitory effect of iberverin (Figure 1A) on different HCC cells, seven HCC cell lines, HCCLM3, HepG2, Huh1, Huh7, Huh7.5.1, SMMC7721 and SNU739 were employed. For a short-term study, HCC cells were treated with the indicated concentration of iberverin and DMSO for 48 h, respectively, and then subjected to the MTT assay. Generally, the antiproliferative effect of iberverin on HCC cells was positively correlated to the dose of iberverin (Figure 1B). Among these HCC cell lines, Huh7, Huh7.5.1, and SNU739 were most sensitive to ibervein with IC_50_ less than 25 μmol/L, followed by SMMC7721 and Huh1 with IC_50_ between 50 and 100 μmol/L (Figure 1B). Therefore, Huh7, Huh7.5.1, and SNU739 cell lines, on which iberverin exerted a relatively strong inhibitory effect, were chosen for further study. As expected, clonogenic survival rate of Huh7, Huh7.5.1 and SNU739 cells was significantly decreased in a dose-dependent manner after iberverin treatment, as indicated by the significant reduction of colony numbers and sizes (Figure 1C).

### 3.2 Iberverin suppresses the migration and invasion of HCC cells

**FIGURE 2:**
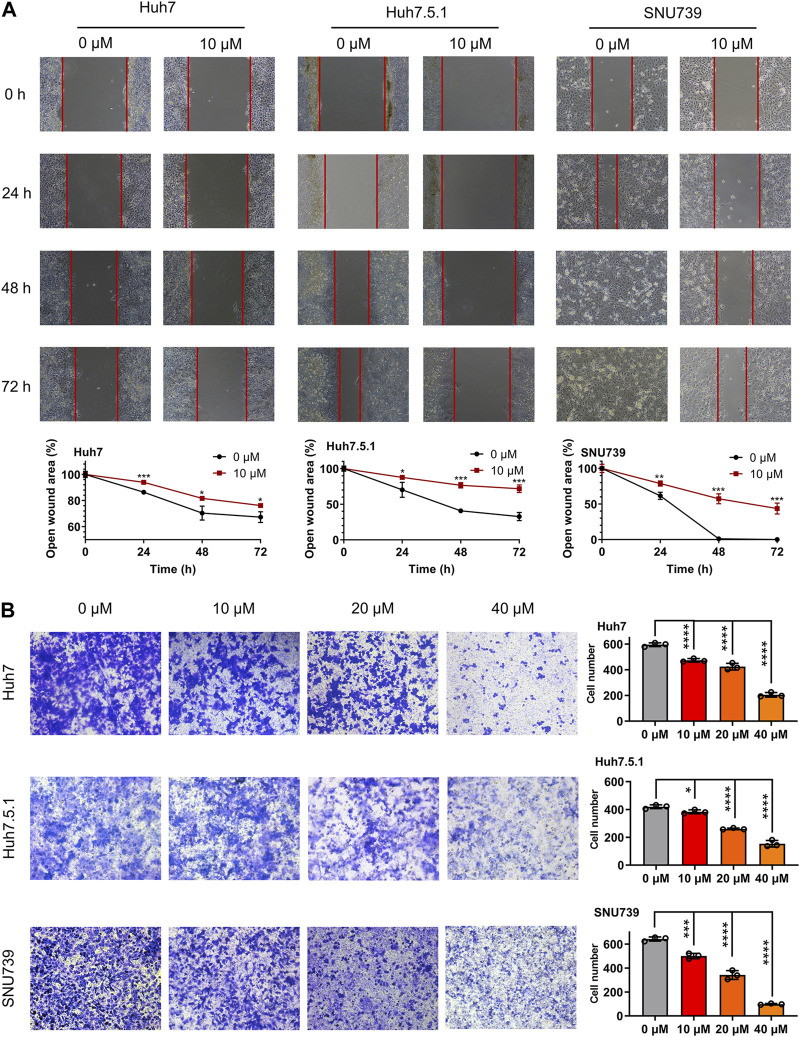
Iberverin suppresses the migration and invasion of HCC cells. **(A)** Wound healing migration assay of Huh7, huh7.5.1 and SNU739 cells incubated with 10 μmol/L iberverin (right panel) and DMSO control (left panel), respectively, for 0, 24, 48, and 72 h (*n* = 3). The relative cell-free gap was evaluated and quantified. **(B)** Transwell invasion assay of Huh7, Huh7.5.1 and SNU739 cells incubated with 10, 20, and 40 μmol/L iberverin or DMSO control for 12 h (*n* = 3). The migrated cells were calculated. Data were shown as mean ± SD. *p < 0.05, **p < 0.01, ***p < 0.001 and ****p < 0.0001.

In order to investigate whether iberverin could impair the migration of HCC cells, wound healing migration assay was conducted. After treatment with 10 μmol/L iberverin, the migration rate of Huh7, Huh7.5.1 and SNU739 cells was remarkably delayed compared with the control group (Figure 2A). Consistent with the result of wound healing migration assay, the invasive ability of Huh7, Huh7.5.1 and SNU739 cells was markedly suppressed in a dose-dependent manner (Figure 2B).

### 3.3 Iberverin induces mitochondrial-related apoptosis in HCC cells

**FIGURE 3:**
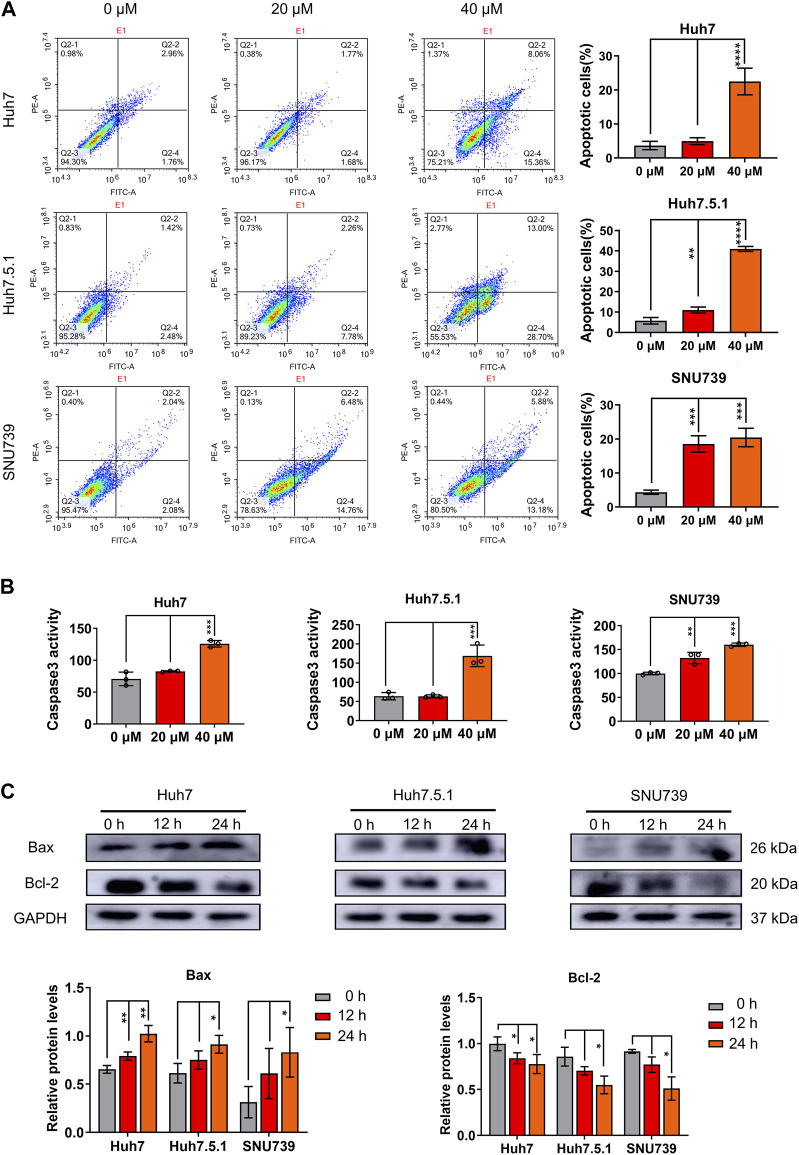
Iberverin induces apoptosis in HCC cells. **(A)** Annexin V-FITC/PI double-staining assay by flow cytometry analysis of apoptosis in Huh7, Huh7.5.1 and SNU739 cells incubated with 20 and 40 μmol/L iberverin or DMSO control for 12 h. The percentage of apoptotic cells was shown on right panel (*n* = 3). **(B)** Determination of Caspase-3 activity in Huh7, Huh7.5.1 and SNU739 cells incubated with 20 and 40 μmol/L iberverin or DMSO control for 12 h (*n* = 3). **(C)** Western blot analysis of apoptotic proteins Bax and Bcl-2 in Huh7, Huh7.5.1 and SNU739 cells incubated with 20 μmol/L iberverin or DMSO control for 12 and 24 h. The histogram represented the expression levels of Bax and Bcl-2 proteins in three independent experiments (*n* = 3). Data were shown as mean ± SD. *p < 0.05, **p < 0.01, ***p < 0.001 and ****p < 0.0001.

To evaluate the role of apoptosis in the growth-inhibitory effects of iberverin in HCC cells, the apoptotic cell death induced by iberverin was characterized by FCM with Annexin V-FITC/PI staining. The number of apoptotic cells in Huh7, Huh7.5.1 and SNU739 cells increased significantly by 7.4, 7.1 and 4.7 times after treatment with 40 μmol/L iberverin, respectively, compared with control cells (Figure 3A). Meanwhile, the effects of iberverin in apoptosis induction of HCC cells is dose-dependent (Figure 3A). Subsequently, a substantial increase in Caspase-3 activity in Huh7, Huh7.5.1 and SNU739 cells was determined after co-incubation with iberverin (Figure 3B). Furthermore, Western blot analysis provided additional supportive evidence, revealing that iberverin enhanced the level of apoptotic protein Bax but repressed the expression of Bcl-2 in Huh7, Huh7.5.1 and SUN739 cells in a time-dependent manner (Figure 3C), confirming the pro-apoptotic effect of iberverin *in vitro*.

**FIGURE 4:**
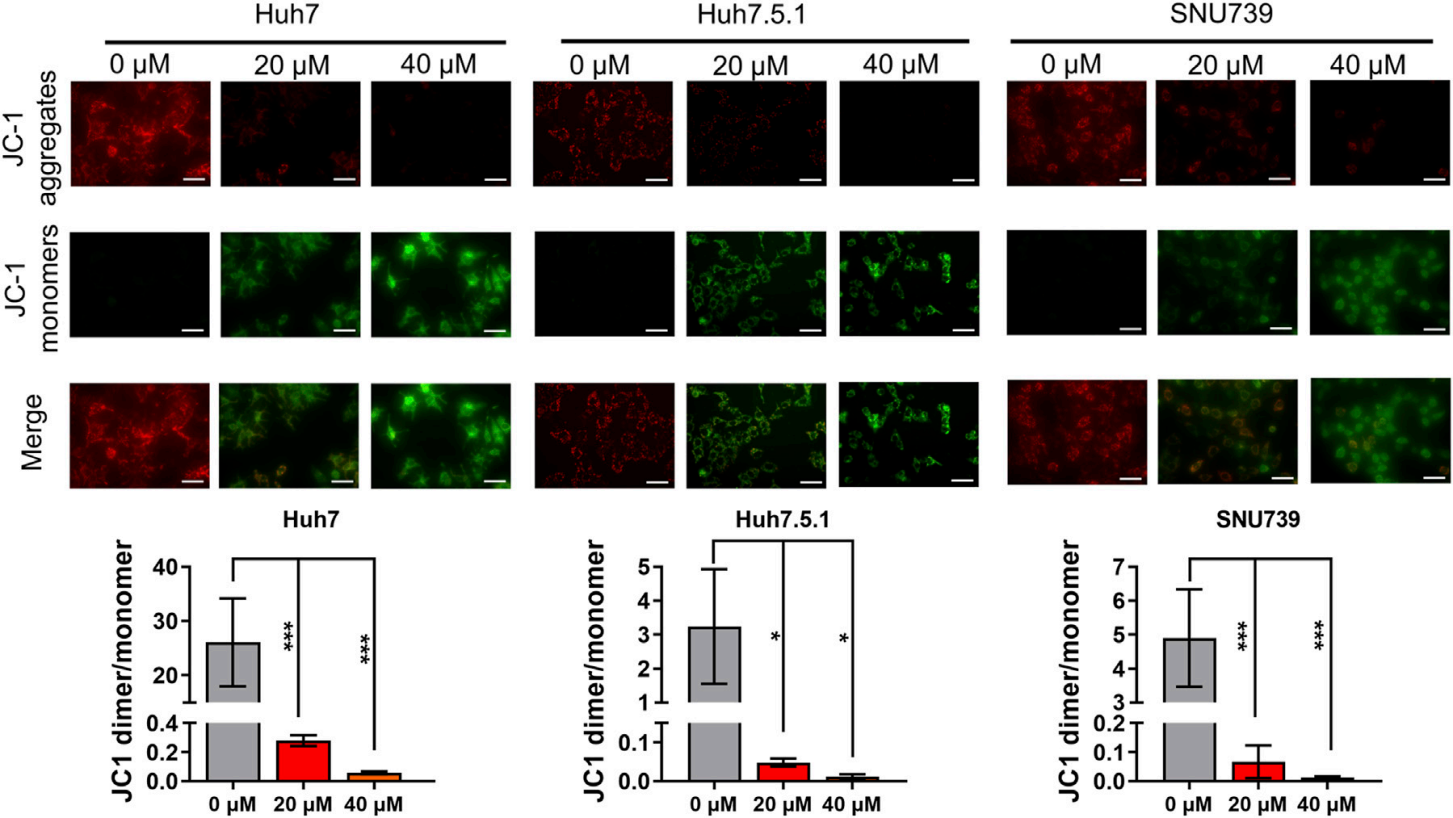
Iberverin causes decreased mitochondrial membrane potential (MMP) in HCC cells. JC-1-based immunofluorescence assay of Huh7, Huh7.5.1 and SNU739 cells incubated with 20 and 40 μmol/L iberverin or DMSO control for 12 h (*n* = 3). Red represents JC-1 aggregate signal, and green represent JC-1 monomer signal. The histogram represented the ratio of JC-1 aggregate/monomer in three independent experiments (*n* = 3). Data were shown as mean ± SD. *p < 0.05 and ***p < 0.001. Scale bars = 50 μm.

As mitochondrial-related apoptosis is triggered by Bcl-2 family-mediated mitochondrial out membrane permeabilization (MOMP), which subsequently activates caspase proteases and ultimately promotes cell self-destruction (Liu et al., 2018), thus, we further determined the changes in MMP. Immunofluorescence assay showed a significant decrease of MMP in all three HCC cell lines treated with iberverin, as indicated by the reduction in the red JC-1 aggregate signal and simultaneously the increase in the green fluorescence intensity (Figure 4).

### 3.4 Iberverin suppresses the growth of HCC xenograft tumors

**FIGURE 5:**
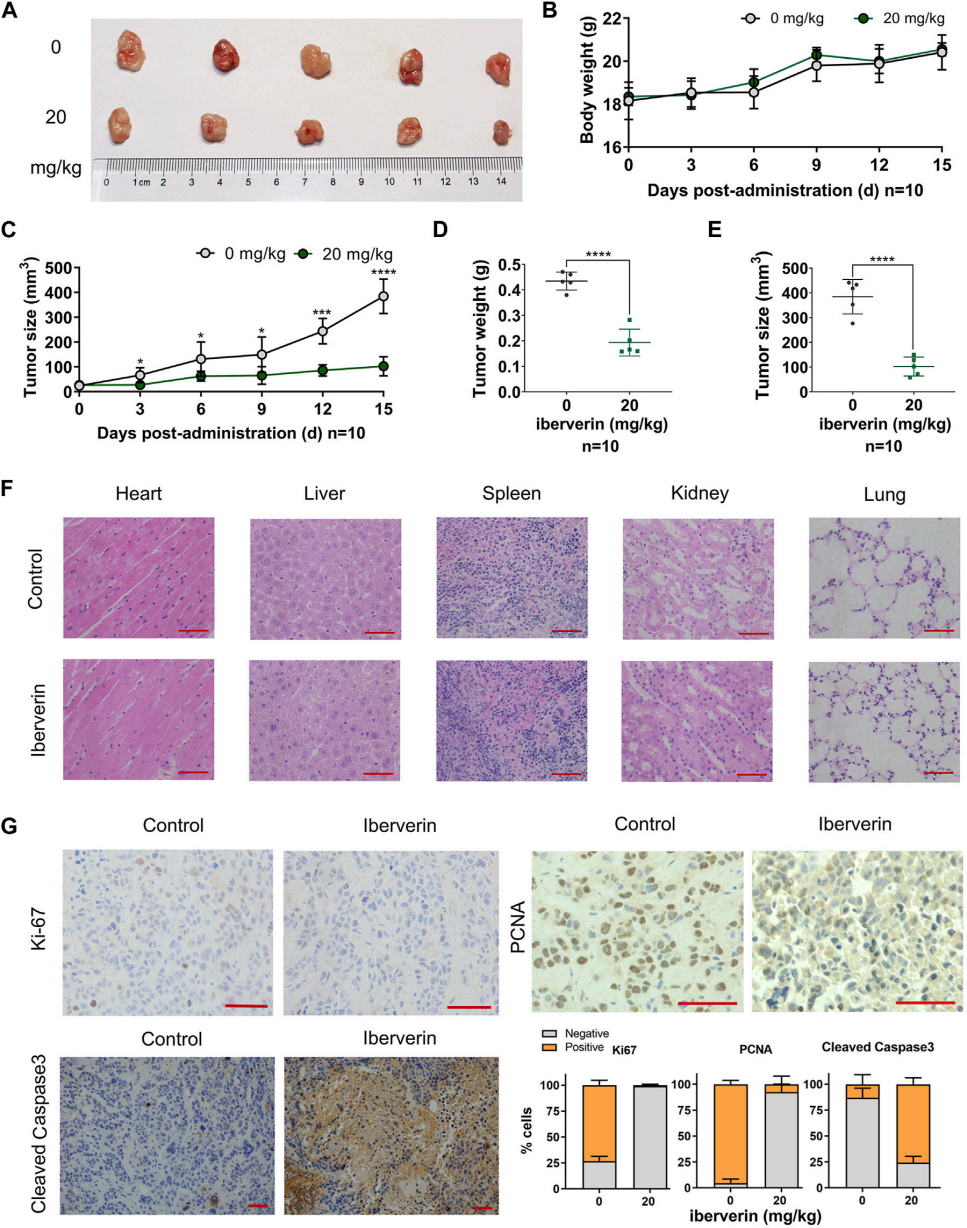
Iberverin inhibits the growth of Huh7.5.1 xenograft tumors in nude mice. **(A)** Huh7.5.1 xenograft tumors from the mice treated with iberverin and DMSO, respectively (*n* = 10). **(B)** The body weight of mice bearing Huh7.5.1 xenograft tumors measured every 3 days. **(C)** The growth curves of xenograft tumors. **(D)** and **(E)** The weight and size of Huh7.5.1 xenografts tumors calculated after executing the nude mice. **(F)** H&E stained tissue sections from mice treated with iberverin and DMSO, respectively. **(G)** Representative IHC staining images of Ki-67, PCNA and cleaved Caspase-3 in Huh7.5.1 xenografts tumors from mice treated with iberverin and DMSO, respectively. Data were shown as mean ± SD. *p < 0.05, ***p < 0.001 and ****p < 0.0001. Scale bars = 50 μm.

To determine the antineoplastic potential of iberverin on HCC *in vivo*, Huh7.5.1 cells were subcutaneously injected into immunodeficient BALB/c nude mice to generate xenograft tumors. These mice were randomly divided into two groups and further subjected to drug regimes. Compared with control group, the marked reduction in the size by 73.4% and weight by 55.3% of Huh7.5.1 xenograft tumors treated with iberverin was detected, respectively (Figures 5A,C–E). However, the body weight of the two groups of mice gradually increased, without significant difference between each other (Figure 5B). In addition, H&E staining of tissue sections (heart, liver, spleen, kidney and lung) from the mice bearing Huh7.5.1 xenograft tumors was also consistent with this observation, exhibiting no obvious histological changes (Figure 5F). These findings confirm that iberverin exerts notably antineoplastic activity against HCC but possesses no systematic toxicity.

To further assess the antiproliferative and pro-apoptotic effect of iberverin on HCC *in vivo*, the expression of Ki-67 and PCNA (two proliferation markers) and cleaved Caspase-3 (an apoptosis marker) in xenograft tumors was investigated by IHC staining. As shown in Figure 5G, both Ki-67 and PCNA were significantly decreased in iberverin-treated Huh7.5.1 xenograft tumors, whereas cleaved Caspase-3 was remarkably increased, suggesting that iberverin could inhibit tumor growth of HCC by suppressing cell proliferation and promoting cell apoptosis *in vivo*.

### 3.5 Transcriptome assay suggests that iberverin activates the G2/M checkpoint and p53 pathway in HCC cells

**FIGURE 6:**
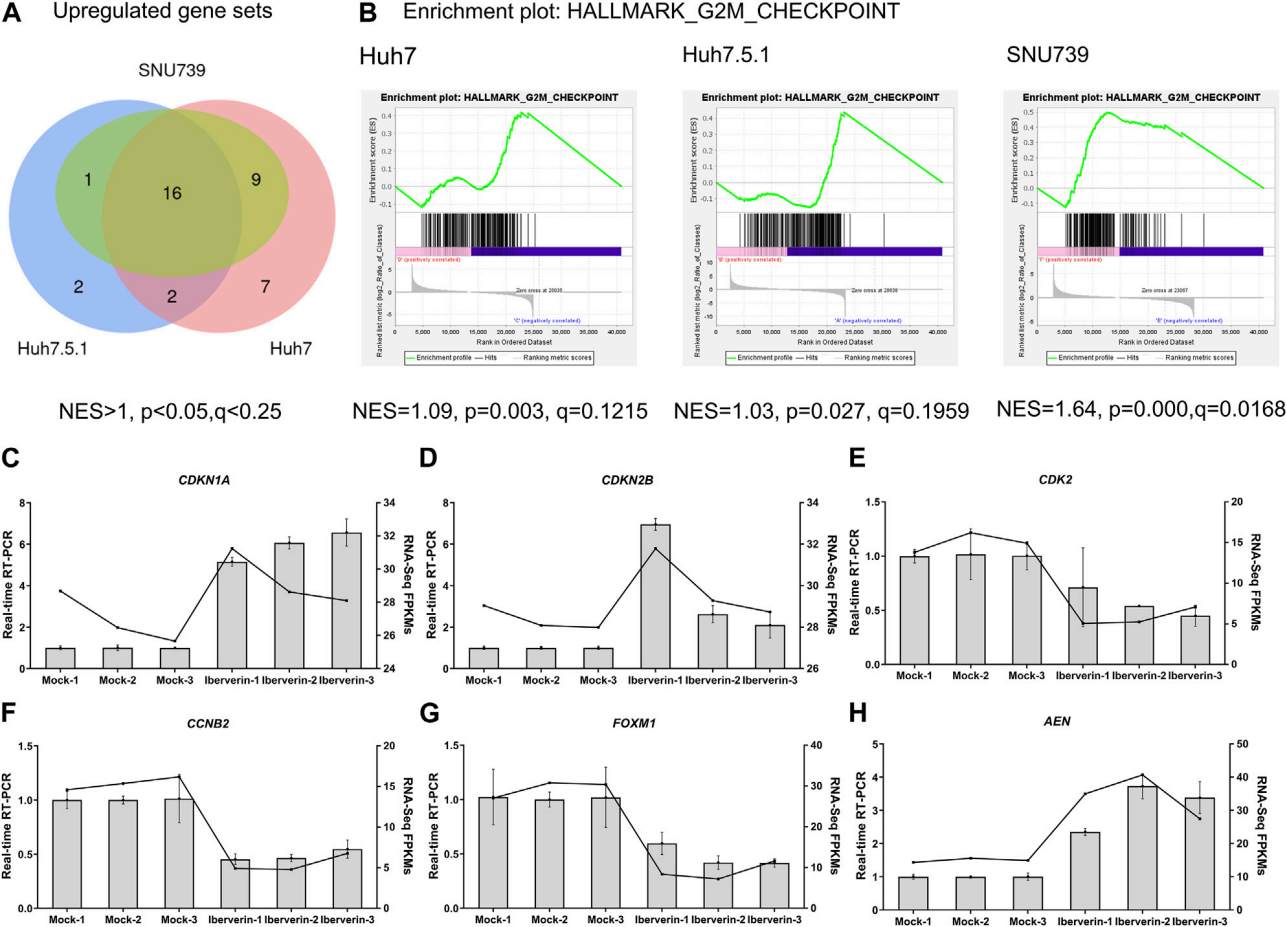
Transcriptome sequencing analysis of HCC cells incubated with or without iberverin. **(A)** Gene set enrichment analysis (GSEA) of the activated gene sets in Huh7, Huh7.5.1 and SNU739 cells after iberverin treatment. Sixteen common activated gene sets were analyzed by Venn diagram. *NES* > 1.0, *p* < 0.05, *q* < 0.25. **(B)** GSEA revealed that G2/M checkpoint gene set was enriched in all 3 cell lines treated with iberverin. **(C–H)** Relative expression of genes associated with cell cycle and p53 signaling pathway in Huh7.5.1 cells treated with iberverin or DMSO.

To further delineate the potential molecular mechanisms underlying the effects of iberverin on HCC, the transcriptome sequencing was conducted to assess the genome-wide effect of iberverin on gene expression in Huh7, Huh7.5.1 and SNU739 cells incubated with ibervern and DMSO, respectively (Supplementary Table S2). GSEA analysis showed that sixteen gene sets were significantly enriched in the iberverin-upregulated transcripts in all 3 cell lines, including genes associated with the G2/M checkpoint, DNA repair and the p53 signaling pathway (Figures 6A,B; Table 1).

**TABLE 1:** The gene sets significantly enriched in HCC cell lines after the treatment of iberverin based on GSEA.

Cell lines	Hallmark gene set names	ES	NES	NOM p-val	FDR q-val
Huh7	HALLMARK_MYC_TARGETS_V2	0.55	1.37	0.001	0.1110
	HALLMARK_UNFOLDED_PROTEIN_RESPONSE	0.53	1.36	0.000	0.0630
	HALLMARK_TNFA_SIGNALING_VIA_NFKB	0.50	1.30	0.000	0.0654
	HALLMARK_MYC_TARGETS_V1	0.46	1.19	0.000	0.1223
	HALLMARK_OXIDATIVE_PHOSPHORYLATION	0.44	1.14	0.000	0.1428
	HALLMARK_REACTIVE_OXYGEN_SPECIES_PATHWAY	0.46	1.13	0.042	0.1300
	HALLMARK_PROTEIN_SECRETION	0.44	1.13	0.005	0.1147
	HALLMARK_MTORC1_SIGNALING	0.43	1.12	0.001	0.1096
	HALLMARK_G2M_CHECKPOINT	0.42	1.09	0.003	0.1215
	HALLMARK_CHOLESTEROL_HOMEOSTASIS	0.43	1.09	0.040	0.1121
	HALLMARK_INTERFERON_ALPHA_RESPONSE	0.42	1.08	0.025	0.1084
	HALLMARK_E2F_TARGETS	0.41	1.08	0.004	0.1039
	HALLMARK_APOPTOSIS	0.41	1.07	0.008	0.1001
	HALLMARK_APICAL_JUNCTION	0.41	1.07	0.005	0.0943
	HALLMARK_UV_RESPONSE_DN	0.41	1.07	0.007	0.0911
	HALLMARK_ADIPOGENESIS	0.41	1.07	0.002	0.0876
	HALLMARK_EPITHELIAL_MESENCHYMAL_TRANSITION	0.41	1.06	0.006	0.0982
	HALLMARK_KRAS_SIGNALING_UP	0.41	1.06	0.007	0.0960
	HALLMARK_INTERFERON_GAMMA_RESPONSE	0.41	1.06	0.006	0.0931
	HALLMARK_UV_RESPONSE_UP	0.41	1.05	0.019	0.0959
	HALLMARK_DNA_REPAIR	0.41	1.05	0.019	0.0923
	HALLMARK_ESTROGEN_RESPONSE_EARLY	0.40	1.05	0.007	0.0939
	HALLMARK_ESTROGEN_RESPONSE_LATE	0.40	1.05	0.016	0.0923
	HALLMARK_GLYCOLYSIS	0.40	1.05	0.008	0.0915
	HALLMARK_HYPOXIA	0.40	1.05	0.009	0.0886
	HALLMARK_FATTY_ACID_METABOLISM	0.40	1.04	0.028	0.0878
	HALLMARK_MYOGENESIS	0.40	1.04	0.016	0.0883
	HALLMARK_HEME_METABOLISM	0.40	1.04	0.017	0.0881
	HALLMARK_ALLOGRAFT_REJECTION	0.40	1.04	0.020	0.0885
	HALLMARK_P53_PATHWAY	0.40	1.04	0.016	0.0881
	HALLMARK_IL2_STAT5_SIGNALING	0.40	1.03	0.034	0.1068
	HALLMARK_XENOBIOTIC_METABOLISM	0.40	1.03	0.039	0.1055
	HALLMARK_COMPLEMENT	0.40	1.03	0.036	0.1045
	HALLMARK_MITOTIC_SPINDLE	0.39	1.02	0.049	0.1234
Huh7.5.1	HALLMARK_TNFA_SIGNALING_VIA_NFKB	0.60	1.42	0.000	0.0475
	HALLMARK_INFLAMMATORY_RESPONSE	0.52	1.23	0.000	0.1355
	HALLMARK_TGF_BETA_SIGNALING	0.50	1.13	0.041	0.2320
	HALLMARK_MYC_TARGETS_V2	0.49	1.12	0.040	0.1908
	HALLMARK_EPITHELIAL_MESENCHYMAL_TRANSITION	0.47	1.11	0.001	0.1635
	HALLMARK_MYC_TARGETS_V1	0.46	1.09	0.001	0.1725
	HALLMARK_DNA_REPAIR	0.46	1.09	0.007	0.1605
	HALLMARK_MTORC1_SIGNALING	0.46	1.08	0.001	0.1497
	HALLMARK_OXIDATIVE_PHOSPHORYLATION	0.45	1.06	0.002	0.1713
	HALLMARK_BILE_ACID_METABOLISM	0.45	1.05	0.043	0.1910
	HALLMARK_HYPOXIA	0.44	1.05	0.010	0.1980
	HALLMARK_ESTROGEN_RESPONSE_EARLY	0.44	1.04	0.015	0.2143
	HALLMARK_MYOGENESIS	0.44	1.04	0.020	0.1971
	HALLMARK_APICAL_JUNCTION	0.44	1.03	0.021	0.1897
	HALLMARK_G2M_CHECKPOINT	0.44	1.03	0.027	0.1959
	HALLMARK_E2F_TARGETS	0.44	1.03	0.032	0.1787
	HALLMARK_APOPTOSIS	0.44	1.03	0.048	0.1743
	HALLMARK_ADIPOGENESIS	0.44	1.03	0.032	0.1723
	HALLMARK_IL2_STAT5_SIGNALING	0.44	1.03	0.040	0.1673
	HALLMARK_P53_PATHWAY	0.44	1.03	0.044	0.1687
	HALLMARK_HEME_METABOLISM	0.43	1.02	0.048	0.1797
SNU739	HALLMARK_MYC_TARGETS_V2	0.65	2.01	0.000	0.0010
	HALLMARK_TNFA_SIGNALING_VIA_NFKB	0.57	1.88	0.000	0.0025
	HALLMARK_G2M_CHECKPOINT	0.50	1.64	0.000	0.0168
	HALLMARK_MYC_TARGETS_V1	0.45	1.49	0.000	0.0375
	HALLMARK_PROTEIN_SECRETION	0.46	1.47	0.004	0.0354
	HALLMARK_TGF_BETA_SIGNALING	0.44	1.35	0.021	0.0622
	HALLMARK_OXIDATIVE_PHOSPHORYLATION	0.40	1.33	0.001	0.0605
	HALLMARK_REACTIVE_OXYGEN_SPECIES_PATHWAY	0.42	1.30	0.040	0.0629
	HALLMARK_E2F_TARGETS	0.39	1.28	0.000	0.0620
	HALLMARK_UNFOLDED_PROTEIN_RESPONSE	0.39	1.27	0.004	0.0619
	HALLMARK_ADIPOGENESIS	0.36	1.18	0.003	0.0969
	HALLMARK_P53_PATHWAY	0.35	1.16	0.000	0.0974
	HALLMARK_HYPOXIA	0.35	1.14	0.002	0.0994
	HALLMARK_MITOTIC_SPINDLE	0.35	1.14	0.003	0.0978
	HALLMARK_EPITHELIAL_MESENCHYMAL_TRANSITION	0.34	1.13	0.003	0.0948
	HALLMARK_MTORC1_SIGNALING	0.34	1.13	0.003	0.0942
	HALLMARK_APICAL_JUNCTION	0.34	1.10	0.003	0.1079
	HALLMARK_UV_RESPONSE_DN	0.34	1.10	0.018	0.1022
	HALLMARK_GLYCOLYSIS	0.33	1.10	0.004	0.1014
	HALLMARK_UV_RESPONSE_UP	0.33	1.09	0.025	0.1059
	HALLMARK_KRAS_SIGNALING_UP	0.33	1.08	0.013	0.1002
	HALLMARK_DNA_REPAIR	0.33	1.06	0.029	0.1073
	HALLMARK_IL2_STAT5_SIGNALING	0.32	1.06	0.021	0.1122
	HALLMARK_MYOGENESIS	0.32	1.05	0.030	0.1147
	HALLMARK_HEME_METABOLISM	0.32	1.04	0.022	0.1196
	HALLMARK_XENOBIOTIC_METABOLISM	0.31	1.03	0.043	0.1461

NES >1, NOM p-val <0.05, FDR q-val <0.25.

Real-time RT-PCR further confirmed that the expression levels of the cell cycle genes, *CDKN1A*, *CDKN2B*, *CDK2*, *CCNB2,* and *FOXM1*, were dysregulated in Huh7.5.1 cells following ibervern exposure (Figures 6C–G). In addition, the expression of *AEN*, which is targeted and induced by p53 and required for efficient DNA fragmentation in p53-dependent apoptosis (Kawase et al., 2008), was also enhanced after ibervern treatment (Figure 6H). These results showed that the cell cycle- and p53-related gene sets could be activated by iberverin, and G2/M DNA damage checkpoint might be a key tumor suppressor checkpoint, indicating the potential regulatory mechanisms of iberverin in terms of its anti-HCC effect.

### 3.6 Iberverin-induced DNA damage causes G2/M cell cycle arrest in a ROS-dependent manner in HCC cells

**FIGURE 7:**
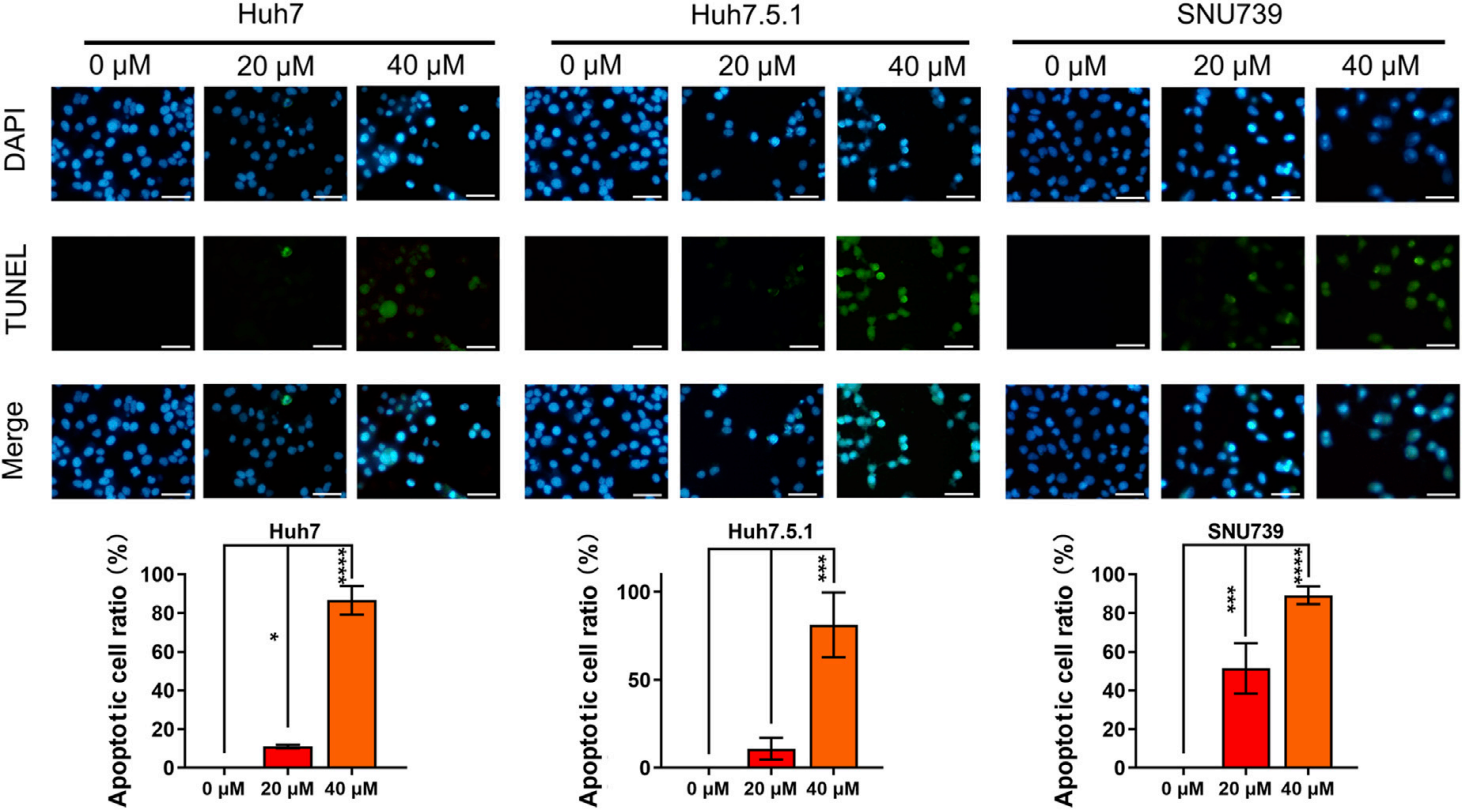
Iberverin induces DNA damage in HCC cells. DAPI staining and TUNEL staining assay of Huh7, Huh7.5.1 and SNU739 cells incubated with 20 and 40 μmol/L iberverin or DMSO control for 12 h (*n* = 3). Blue and green fluorescence represented nuclei and DNA fragmentation, respectively. DNA-damaged nuclei were indicated in the merged images. The histogram represented the percentage of the apoptotic cells with DNA-damaged nuclei in three independent experiments (*n* = 3). Data were shown as mean ± SD. *p < 0.05, ***p < 0.001 and ****p < 0.0001. Scale bars = 50 μm.

Based on GESA, DNA repair gene set was significantly enriched in all 3 cell lines (Table 1), indicating that iberverin might induce DNA damage. As DNA damage, most often involving large DNA fragmentation, is widely observed in dying cells that display apoptotic morphological changes (Kaufmann et al., 2000), a DAPI staining and TUNEL staining assay was conducted to examine iberverin-induced DNA damage in HCC cells. It was revealed that iberverin induced a marked increase of green fluorescence in HCC cells in a dose-dependent manner (Figure 7), indicating the occurrence of DNA damage in the nuclei. What was more, when treated with iberverin at a concentration of 40 μmol/L, a large number of nuclei had broken down, as reflected by the DAPI staining (Figure 7). These results clearly indicated that iberverin exposure induced DNA damage in HCC cells.

**FIGURE 8:**
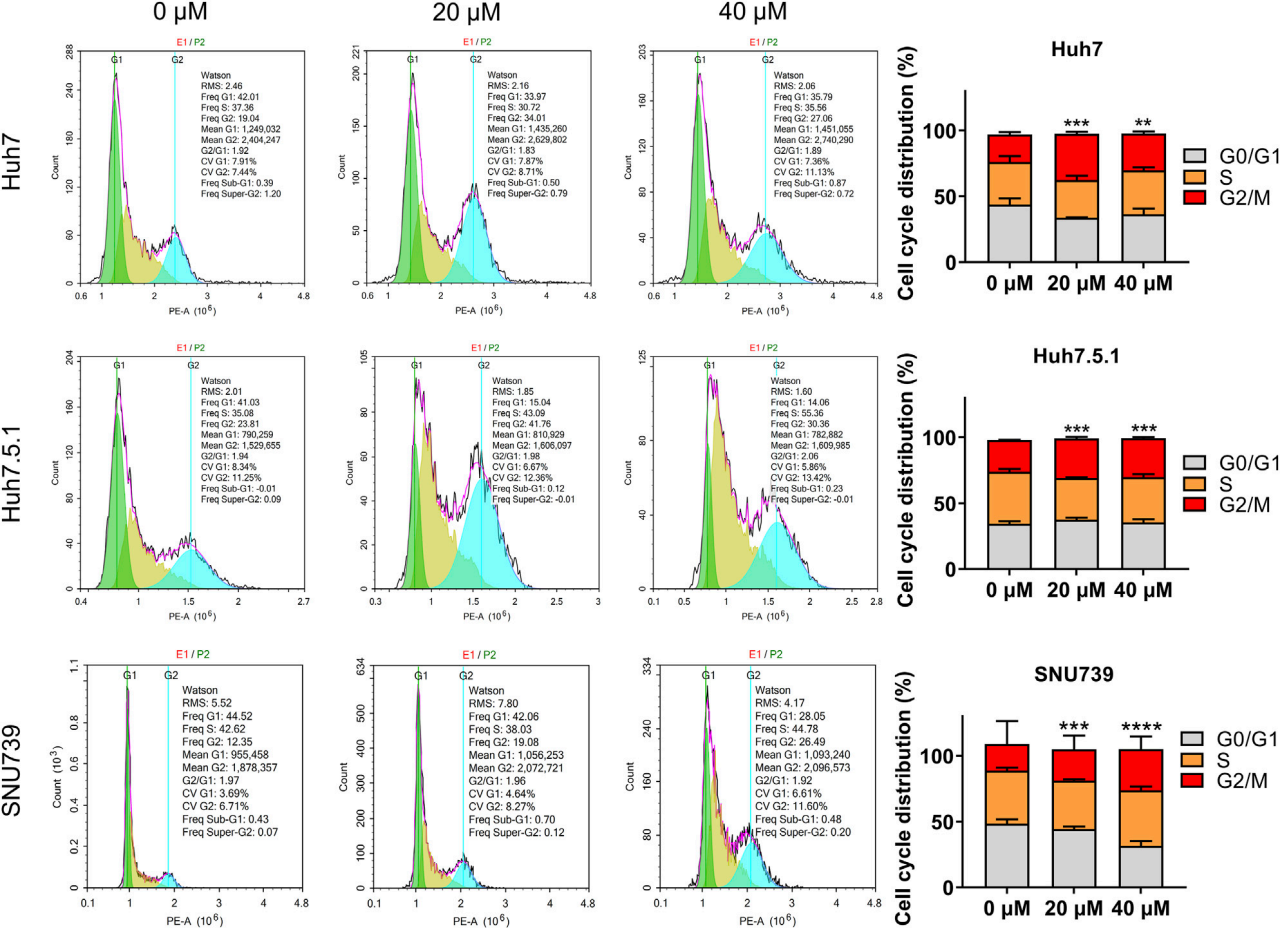
Iberverin induces cell cycle arrest in G2/M phase in the proliferation of HCC cells. The cell cycle distribution was determined in Huh7, Huh7.5.1, and SNU739 cells incubated with 20 and 40 μmol/L iberverin or DMSO for 12 h by flow cytometry analysis. The percentage of the G1, S, G2/M phases was evaluated and shown on right panel (*n* = 3). Data were shown as mean ± SD. **p < 0.01, ***p < 0.001 and ****p < 0.0001.

As DNA damage can cause cell cycle arrest, we further investigated the implication of iberverin in cell cycle distribution through PI/RNase staining by FCM analysis. The result revealed that iberverin obviously induced G2/M cell cycle arrest in Huh7, Huh7.5.1 and SNU739 cells, whereas reduced the population of cells in G1 and S phases (Figure 8). Moreover, the core enrichment genes for the activated G2/M checkpoint from GSEA were further investigated based on Reactome pathway analysis, and fourteen genes were finally identified in all 3 cell lines (Table 2). In this study, p53 pathway, which has been demonstrated to be enhanced by DNA damage and regulate the cell cycle progression (Helton and Chen, 2007; Engeland, 2022), was also activated based on GSEA (Table 1). All these results suggested that the inhibitory roles of iberverin in the growth of HCC cells could be attributed to G2/M arrest caused by DNA damage.

**TABLE 2:** The core enrichment genes of hallmark G2M CHECKPOINT gene set in HCC cell lines treated with iberverin based on GSEA.

Cell lines	Genes^a^	Rank metric scores	Running ES	Core enrichment
Huh7	HUS1*	0.674280345	−0.066083774	Yes
	CDC25A*	0.54730624	−0.021954276	Yes
	*H2BC12*	0.397074491	0.007916474	Yes
	CDC6*	0.246321559	0.03360164	Yes
	ORC6*	0.231656134	0.035954203	Yes
	DBF4*	0.189768985	0.0422332	Yes
	EXO1*	0.116855599	0.047004547	Yes
	RPA2*	0.054428279	0.038238443	Yes
	ORC5*	0.030267492	0.031266432	Yes
	MCM6*	−0.037066828	−0.002669783	Yes
	CHEK1*	−0.096802443	−0.013186244	Yes
	*MCM2*	−0.223403722	0.007531589	Yes
	*NSD2*	−0.247092977	0.014205299	Yes
	*MCM5*	−0.358161062	0.079927996	Yes
	CDC7*	−0.361887842	0.0952116	Yes
	MCM3*	−0.531770051	0.21273686	Yes
	*CCNB2*	−0.537943661	0.22775517	Yes
	WRN*	−0.545878589	0.2426727	Yes
	CDK1*	−0.618214726	0.28907275	Yes
	*CDC45*	−0.691384554	0.3269976	Yes
	*BARD1*	−0.762358725	0.38143694	Yes
Huh7.5.1	HUS1*	0.660314441	−0.09981071	Yes
	CDC25A*	0.376548588	−0.070587374	Yes
	ORC6*	0.309317529	−0.06927699	Yes
	WRN*	0.283825129	−0.07110837	Yes
	CDC6*	−0.005761847	−0.11234292	Yes
	CHEK1*	−0.058090433	−0.12541598	Yes
	*MCM5*	−0.141590282	−0.13823974	Yes
	DBF4*	−0.162432313	−0.13973919	Yes
	RPA2*	−0.450888664	−0.1486315	Yes
	EXO1*	−0.461915255	−0.1434403	Yes
	ORC5*	−0.481425107	−0.14327826	Yes
	*MCM2*	−0.571308434	−0.12855072	Yes
	*NSD2*	−0.627520025	−0.09856759	Yes
	*CDC45*	−0.662444711	−0.09363544	Yes
	*H2BC12*	−0.698840201	−0.060440045	Yes
	MCM3*	−0.786862969	−0.017804312	Yes
	MCM6*	−0.997158408	0.08679963	Yes
	CDK1*	−1.127957225	0.11787925	Yes
	*BARD1*	−1.247657418	0.21405476	Yes
	CDC7*	−1.470173478	0.26555622	Yes
	*CCNB2*	−1.603772998	0.30941218	Yes
SNU739	WRN*	0.505983651	0.0102293	Yes
	ORC5*	0.497382909	0.040960204	Yes
	DBF4*	0.401874721	0.088307604	Yes
	CDC25A*	0.346485287	0.15242542	Yes
	CDC6*	0.332688808	0.17050894	Yes
	RPA2*	0.306001008	0.20940134	Yes
	ORC6*	0.299213856	0.22673178	Yes
	EXO1*	0.282092005	0.27787817	Yes
	HUS1*	0.270357847	0.30350763	Yes
	CHEK1*	0.269389927	0.30905035	Yes
	CDK1*	0.252586246	0.31889796	Yes
	MCM3*	0.189492613	0.4019202	Yes
	CDC7*	0.16343157	0.4360845	Yes
	MCM6*	0.13451378	0.46395138	Yes

^a^ Fourteen common core enrichment genes that are present in all three HCC cell lines and associated with G2/M checkpoint based on Reactome pathway analysis are indicated by asterisks.

**FIGURE 9:**
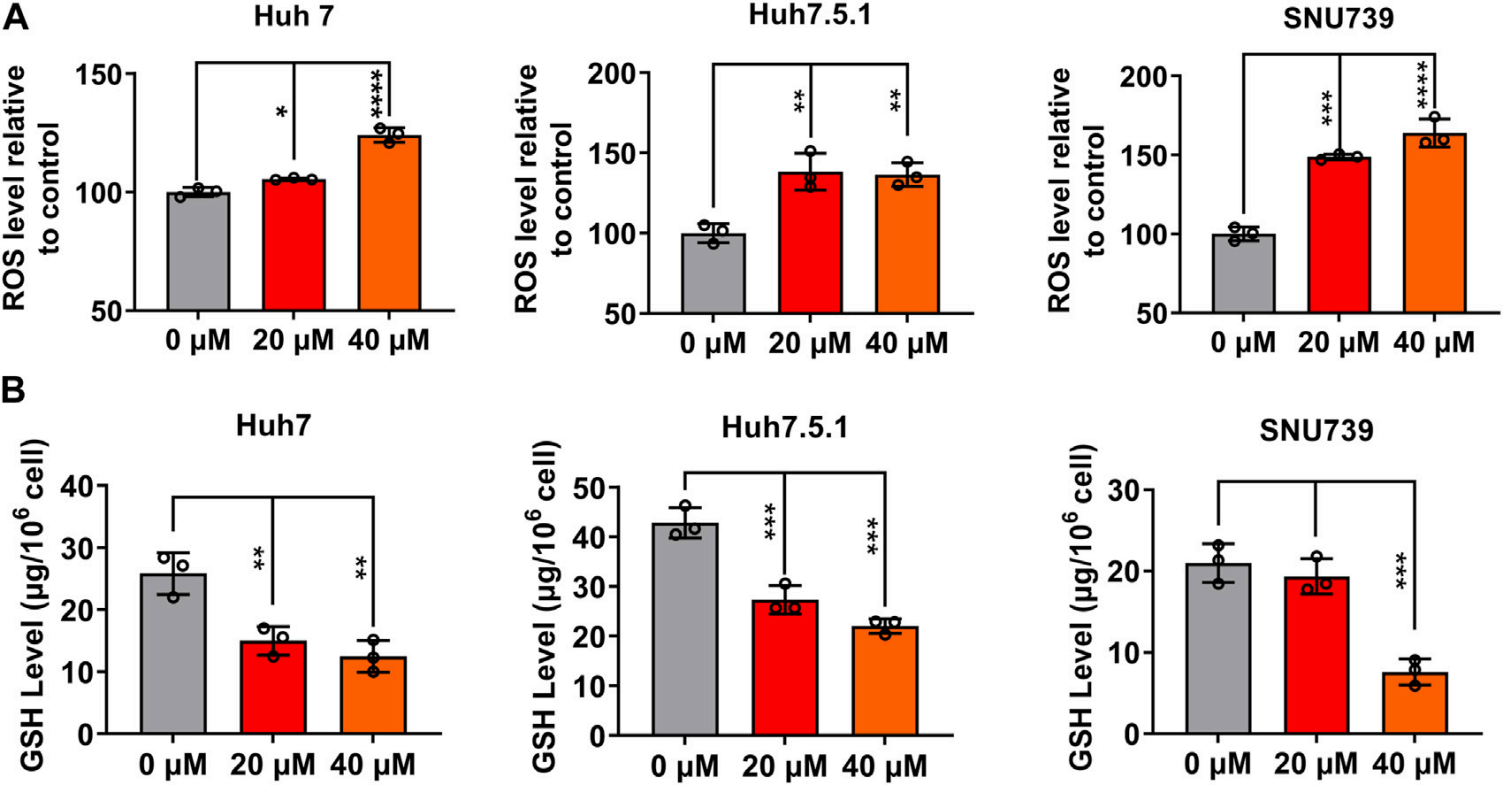
Iberverin enhances oxidative stress in HCC cells. The levels of intracellular reactive oxygen species (ROS) **(A)** and glutathione (GSH) **(B)** were determined in Huh7, Huh7.5.1 and SNU739 cells after incubation with 20 and 40 μmol/L iberverin or DMSO for 12 h (n = 3). Data were shown as mean ± SD. *p < 0.05, **p < 0.01, ***p < 0.001 and ****p < 0.0001.

ROS accumulation and GSH depletion that have been found to promote cancer cell death are crucial events in oxidative stress (Arfin et al., 2021). They have been shown to mediated DNA damage, checkpoint responses, and apoptosis in diverse cancers (Barzilai and Yamamoto, 2004; Guachalla and Rudolph, 2010; Guo et al., 2014; Sadiq, 2023). Here, we investigated the ROS accumulation and GSH depletion in HCC cells, and our results demonstrated that iberverin treatment could enhance oxidative stress in Huh7, Huh7.5.1 and SNU739 cells in a dose-dependent manner, as evidenced by the increase in the production of ROS and simultaneously depletion of GHS (Figure 9). Thus, it was suggested that iberverin caused DNA damage and G2/M checkpoint mediated by ROS generation.

**FIGURE 10:**
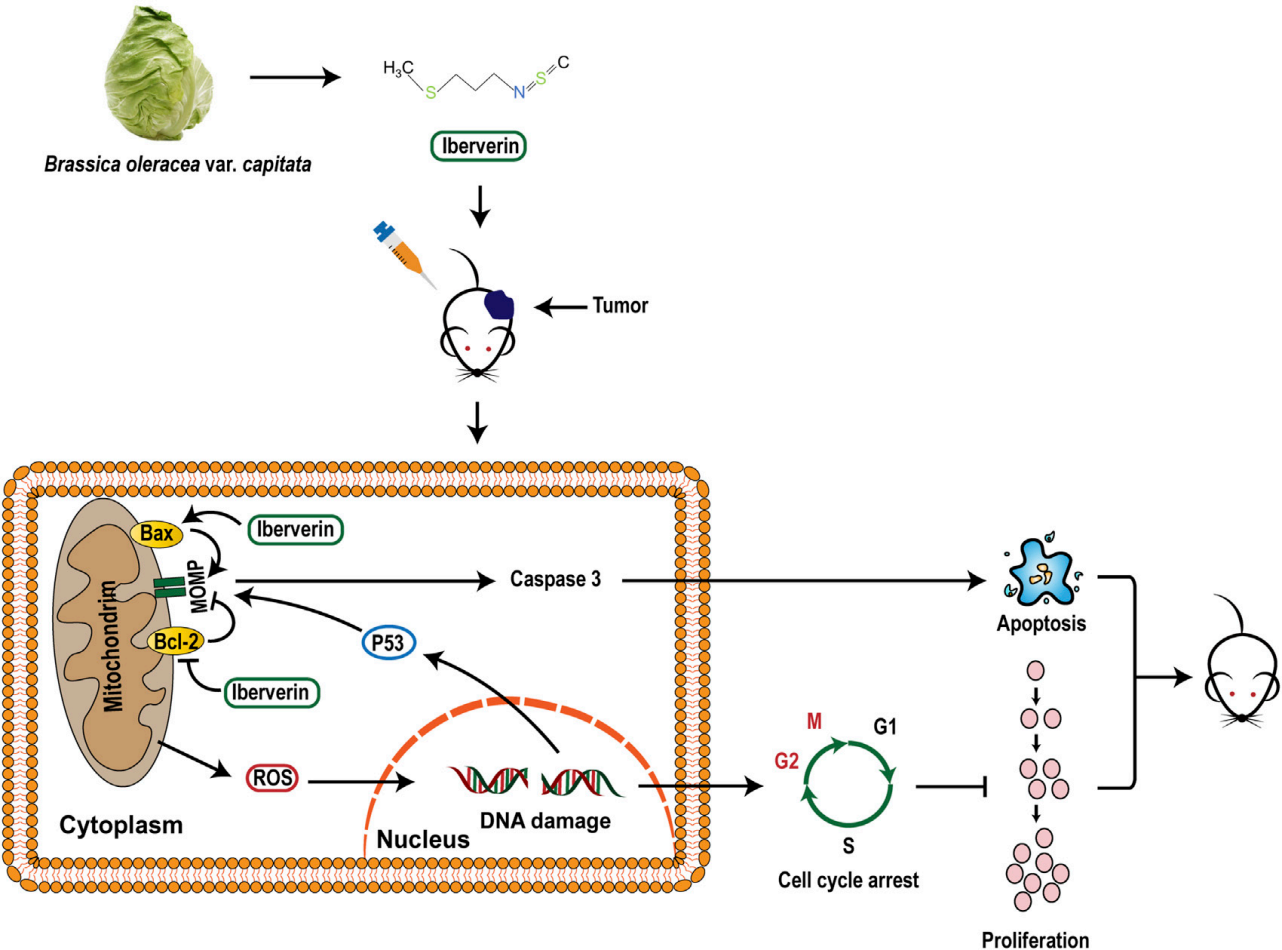
A proposed mechanistic model of the inhibitory effect of iberverin in HCC.

In summary, the present study confirmed that iberverin could induce DNA damage and G2/M arrest through ROS generation and promote mitochondrial-related apoptosis, ultimately exerting anti-HCC activities (Figure 10).

## 4 Discussion

There is growing evidence from encouraging *in vitro* and *in vivo* animal models that ITCs have multiple biological activities including plant defense and benefits to human health (antioxidant, antimicrobial and anticarcinogenic properties) (Jain et al., 2019; Chuang et al., 2020; Li et al., 2021; Lohning et al., 2021; Dixit et al., 2022). ITCs have been found to exert anticarcinogenic activity in diverse cancers, such as lung cancer, breast cancer, prostate cancer and bladder cancer (Psurski et al., 2019; Zheng et al., 2019; Justin et al., 2020; Nguyen et al., 2020). Accumulating studies *in vitro* and *in vivo* animal models revealed that ITCs, such as BITC, PEITC and SFN, could inhibit HCC by suppressing cell proliferation, inhibiting cell migration, inducing apoptosis and/or autophagy (Dinh et al., 2021; Haq et al., 2022; Zhang et al., 2022). As a predominant ITC derived from the seeds of oxheart cabbage, iberverin has been proved to have great antineoplastic activity in lung cancer A549 cells and cervical cancer Hela cells (Wang et al., 2010; Wang et al., 2016). In this study, we dissected the effect of iberverin in human HCC cells *in vitro* and found that iberverin could inhibit cell proliferation, suppress migration and invasion, induce apoptosis, and impair cell cycle progression. In addition, the xenograft tumor assay further verified the antineoplastic roles of iberverin in HCC *in vivo* without obvious toxicity, making it a promising candidate for novel anti-HCC treatments.

Previous studied have reported that ITCs, such as PEITC, BITC and SFN, could trigger a series of mitochondria-related apoptotic responses, which ultimately promote the accumulation of ROS that causes DNA damage to kill cancer cells (Sestili et al., 2010; Huang et al., 2014; Henklewska et al., 2021). Our results showed that iberverin might also exert its anticarcinogenic activity against HCC through mitochondria-related apoptotic pathway. Decreased MMP mediated by Bax and Bcl-2 was observed in HCC cells after iberverin treatment. In addition, GSEA analysis revealed that genes enriched in the tumor suppressor p53 pathway were activated in HCC cells following iberverin exposure, which could also directly affect MMP (Noutsopoulos et al., 2010). Mitochondria-related apoptotic responses induced by iberverin, including decrease of MMP, increase of Bax expression and decrease of Bcl-2 expression, could further promote the enhancement of MOMP, which further activates Caspase-dependent apoptosis and promotes ROS generation that causes DNA damage (Estaquier et al., 2012).

Accumulating evidence from encouraging *in vitro* and *in vivo* animal models has demonstrated that ROS induces DNA damage, checkpoint responses, and apoptosis in diverse cancers (Barzilai and Yamamoto, 2004; Guachalla and Rudolph, 2010; Guo et al., 2014; Sadiq, 2023). In some cases, ITCs cause cell cycle arrest via the enhanced ROS accumulation. For example, a recent study showed that SFN, iberin and Alyssin induced the intracellular ROS generation that led to S and G2/M phase arrest, and ultimately blocked proliferation in HepG2 cells (Ye et al., 2020). In the present study, we also found that iberverin could induce DNA damage and cause G2/M cell cycle arrest in a ROS-dependent manner in HCC, which was further supported by the enrichment gene sets for the activated G2/M checkpoint and DNA repair from GSEA.

In summary, our study demonstrates that iberverin induces the production of ROS, causing DNA damage and subsequent G2/M cell cycle arrest, and activates mitochondrial-related apoptotic responses, which in turn induce growth inhibitory and apoptosis in HCC. Furthermore, our results suggests that G2/M DNA damage checkpoint might be a key tumor suppressor checkpoint and critical for the anti-cancer activity of iberverin. Our data indicate that iberverin is a promising biotherapeutic agent against HCC. In order to maximize the potential clinical application of iberverin, more experimental support from *in vivo* and *in vitro* studies are urgently needed in future.

## Data Availability

The original contributions presented in the study are publicly available. This data can be found here: [http://www.ncbi.nlm.nih.gov/bioproject/1048638. Accession number:PRJNA1048638].
